# Permanganate of Potash in Diabetes

**Published:** 1853-03

**Authors:** 


*Permanganate of Potash in Diabetes.*—A gentleman, upwards of 60, residing in the country, had for several months been suffering from confirmed diabetus mellitus when I first saw him, two years and a half ago. In the course of the twenty-four hours he passed from ten to twelve pints of urine, the specific gravity of which was 1.035 in the morning, and 1.040 in the evening. The patient was greatly emaciated, and he suffered severely from constant pains in the back and thighs, and from increasing debility. After he had perseveringly tried various remedies for nine or ten months, without deriving any benefit, I began, in August, 1850, to give him the permanganate of potash in solution, in doses of two grains, which were afterwards increased to three grains, three times a day. In the course of a few days a visible improvement took place; he felt better; the quantity of urine began to diminish, and the thirst became less troublesome. These favorable symptoms were soon followed by some return of appetite, and a gradual increase of strength. This amendment went on without interruption; the medicine was continued for three months, until the following November, when all medical treatment was discontinued, as the patient felt quite well, and the urine was reduced to its natural amount. A remarkable fact must, however, be mentioned, that at that time, notwithstanding the restoration of the general health, the urine still contained a considerable quantity of sugar. I am not aware whether this state still exists, but the patient's report to me a few days since ran thus: "I pass less than a pint of urine during the night, somewhat more during the day; the pains are gone, and I can walk a fair distance without fatigue." The dose of permanganate of potash which I have generally found to agree best with the stomach, is from one to three grains in solution, and it should be given in three or four tablespoonfuls of water three times a day, shortly before meals.—*Mr. George Sampson in Lancet.*

